# IoTNet: An Efficient and Accurate Convolutional Neural Network for IoT Devices

**DOI:** 10.3390/s19245541

**Published:** 2019-12-14

**Authors:** Tom Lawrence, Li Zhang

**Affiliations:** Department of Computer and Information Sciences, Faculty of Engineering and Environment, Northumbria University, Newcastle upon Tyne NE1 8ST, UK; tom.lawrence@northumbria.ac.uk

**Keywords:** computational complexity, Convolutional Neural Network, computer vision, deep network architecture, efficient architecture, image classification, deep learning

## Abstract

Two main approaches exist when deploying a Convolutional Neural Network (CNN) on resource-constrained IoT devices: either scale a large model down or use a small model designed specifically for resource-constrained environments. Small architectures typically trade accuracy for computational cost by performing convolutions as depth-wise convolutions rather than standard convolutions like in large networks. Large models focus primarily on state-of-the-art performance and often struggle to scale down sufficiently. We propose a new model, namely IoTNet, designed for resource-constrained environments which achieves state-of-the-art performance within the domain of small efficient models. IoTNet trades accuracy with computational cost differently from existing methods by factorizing standard 3 × 3 convolutions into pairs of 1 × 3 and 3 × 1 standard convolutions, rather than performing depth-wise convolutions. We benchmark IoTNet against state-of-the-art efficiency-focused models and scaled-down large architectures on data sets which best match the complexity of problems faced in resource-constrained environments. We compare model accuracy and the number of floating-point operations (FLOPs) performed as a measure of efficiency. We report state-of-the-art accuracy improvement over MobileNetV2 on CIFAR-10 of 13.43% with 39% fewer FLOPs, over ShuffleNet on Street View House Numbers (SVHN) of 6.49% with 31.8% fewer FLOPs and over MobileNet on German Traffic Sign Recognition Benchmark (GTSRB) of 5% with 0.38% fewer FLOPs.

## 1. Introduction

Convolutional Neural Networks (CNNs) have proved revolutionary in computer vision applications and consistently outperform traditional models or even humans at image recognition tasks. CNNs are often benchmarked on computer vision tasks but their impacts are far wider-reaching. Recently, the studies of [1] adopted a CNN model to perform gas identification as part of the wider research area of electronic noses (ENs).

Many studies within computer vision focus on improving accuracy by designing a new state-of-the-art model, typically only ever constrained by the resources available on high-end graphics cards. State-of-the-art models are typically very deep. This is because the network generalization capability is enhanced, as the network goes deeper. However, the downside to deep models is that even the most cutting-edge and efficient state-of-the-art models, such as EfficientNet [2], still contain millions of parameters and billions of FLOPs. Models such as these require significant computational resources to execute, and exhibit diminishing returns when scaling, meaning that a large increase in model size is required to obtain a small improvement of accuracy. Therefore, such an approach results in a very large yet extremely accurate model. When deploying a CNN model within a resource-constrained environment such as IoT devices or smartphones, it becomes critical to find a balance between model accuracy and computational cost to ensure the model will function well within resource limited environments. When finding such a trade-off, two main approaches exist. (1) The first mechanism is to scale down a large model to fit the constraints of the target device as it seems reasonable to assume that if a large increase in the size of a state-of-the-art model results in a small improvement in accuracy, then a large reduction in model size would result in a small loss of performance. While this is true to an extent, the point at which accuracy starts to drop rapidly occurs while the model is still very large. This is because the state-of-the-art accuracy-focused models contain strategies which help such networks overcome the types of issues encountered during training, such as overfitting. To scale such a model down sufficiently enough for tightly constrained environments, expert knowledge and trial-and-error would be required. (2) Alternatively, the second approach is to design models specifically for computational constrained environments. As an example, efficiency-focused models include MobileNet [3], ShuffleNet [4] and EffNet [5]. Such models excel at delivering far greater accuracy than would be possible by significantly scaling down a large model. This is achieved by making design choices which reduce the computational cost, often by performing convolutions as depth-wise separable convolutions instead of normal convolutions employed by their larger model counterparts. A distinction between the above two types of convolutions (i.e., normal and depth-wise separable convolutions) is discussed comprehensively below in Section 1.2. Both of the above two approaches sacrifice model performance in the trade of enhanced computational efficiency.

**Figure 1:**
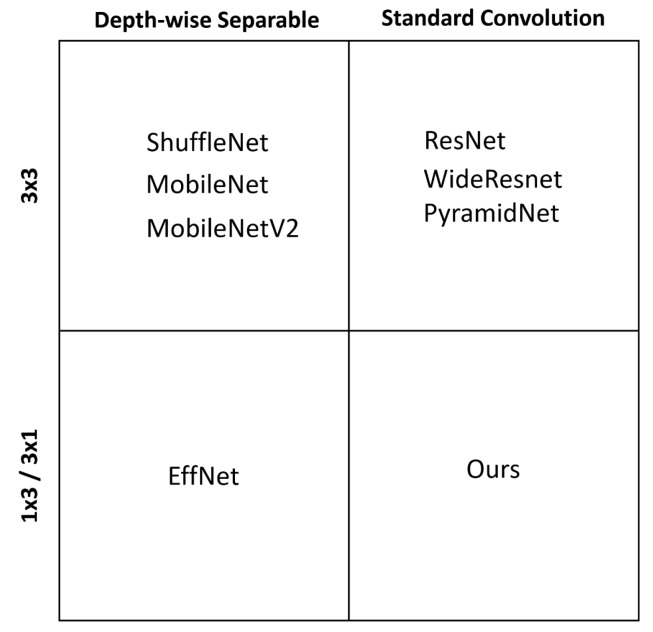
Our model dubbed IoTNet is distinctive from other related works as it uses pairs of 1 × 3 and 3 × 1 standard convolutions, rather than 3 × 3 standard convolutions typically found in large models, or depth-wise separable convolutions used in efficiency-focused models.

Therefore, as compared with the aforementioned methods, the motivation of this research is to design a novel efficiency-focused model specifically for resource-constrained devices which greatly improves accuracy, and reduces computational cost simultaneously. Specifically, we propose a new model, namely IoTNet, which improves the trade-off between accuracy and computational cost by avoiding the common pitfall of efficiency-focused related studies which is to perform convolutions as depth-wise separable operations to reduce computation cost. We instead reduce computation by factorizing the 3 × 3 standard convolutions found in large and highly accurate models into pairs of 1 × 3 and 3 × 1 normal convolutions which reduces the number of parameters by 33%. The empirical results indicate that our approach delivers significantly enhanced performance with less computational cost measured as a reduction in FLOPs. The way our model differs from other existing studies is visualized in Figure 1.

The main contributions of this research are as follows.

- We propose a new architecture, namely IoTNet, which is designed specifically for performance constrained environments such as IoT devices, smartphones or embedded systems. It trades accuracy with a reduction in computational cost differently from existing methods by employing novel pairs of 1 × 3 and 3 × 1 normal convolutions, rather than using depth-wise separable convolutions.
- An in-depth comparison of the proposed architecture against efficiency-focused models including MobileNet [3], MobileNetV2 [6], ShuffleNet [4] and EffNet [5] has been conducted using CIFAR-10 [7], Street View House Numbers (SVHN) [8] and German Traffic Sign Recognition Benchmark (GTSRB) [9] data sets. The empirical results indicate that the proposed block architecture constructed exclusively from pairs of 1 × 3 and 3 × 1 normal convolutions, with average pooling for downsampling, outperforms the current state-of-the-art depth-wise separable convolution-based architectures in terms of accuracy and cost.
- A direct comparison of pairs of 1 × 3 and 3 × 1 normal convolutions against 3 × 3 standard convolutions has also been conducted. The empirical results indicate that our approach results in a more accurate and efficient architecture than a scaled-down large state-of-the-art network.

The impact of this work is to enable a significantly more accurate model to be deployed within resource-constrained environments, which is of great benefit to the wider research community.

### 1.1. Related Work

Works relating to IoT devices identify a real need for more accurate models within resource-constrained environments. Recently, the research studies of [10] highlighted how IoT devices, such as Raspberry Pi, make edge computing a reality, as cheap devices can be interconnected to form network infrastructures. When interconnected, such networks have been used to tackle a range of problems, including pollution, air, water, food, and fire sensing, heartbeat and blood pressure monitoring, and motion tracking. Furthermore, the studies of [11] presented a novel solution to decentralize data exchange based on wireless IoT and blockchain technologies and highlighted how IoT-based solutions have illustrated exponential growth owing to a rise in IoT applications within smart cities and health tracking domains. Because of the fast growth and the range of interesting applications for IoT devices, more accurate and efficient deep learning models are essential. Moreover, a recent case study [12] especially focused on sensor reliability relating to LiDAR sensors with IoT capabilities. Their work pointed out that such types of sensor devices are becoming widespread. Their IoT capable devices employed a range of models to perform tasks such as driver assisting obstacle detection within cars and fault detection, yet more advanced deep learning models could be deployed in such applications providing such networks can deliver sufficient accuracy and efficiency.

Research into architectures which improve the accuracy and performance of CNNs has been an active research area for some time. This work has resulted in notable architectures such as ResNet [13], WideResnet [14], AlexNet [15] and PyramidNet [16]. The 3 × 3 convolution has proven a popular choice for many architectures but Inception-v3 [17] and Inception-v4 [18] have shown that the 3 × 3 convolution can be replaced with a 3 × 1 and 1 × 3 convolution, resulting in a 33% reduction in parameters. While the above variants of the inception block make use of 1 × 3 and 3 × 1 convolutions, the block contains multiple branches, filter concatenations and 1 × 1 convolutions. Multiple branches were proposed within the inception model to train deeper models. The drawback of such a practice is that in resource-constrained environments, models tend to be shallower due to the computational constraints and multiple branches substantially increase the computational cost for a given depth. In comparison with these existing models, the proposed architecture in this research differs from inception networks as it contains one branch, and has no filter concatenation which reduces overhead and does not use 1 × 1 convolutions. All the aforementioned existing models are optimized to achieve state-of-the-art performance. However, the drawback when deployed to resource-constrained environments is that they are typically large and contain additional operations to address overfitting [19]. While it is possible to scale these large models down, they are specifically designed to maximize accuracy and are trained on high-end GPU machines.

Other research efforts in building network architectures suitable for use on performance restricted environments such as IoT and smartphones have led to another category of models, specifically designed to be computationally efficient. State-of-the-art architectures of this type of models include MobileNet [3], MobileNetV2 [6], ShuffleNet [4], LiteNet [20] and EffNet [5].

A comparative analysis of all related studies has been summarized in Table 1, followed by in-depth discussions.

**Figure 2:**
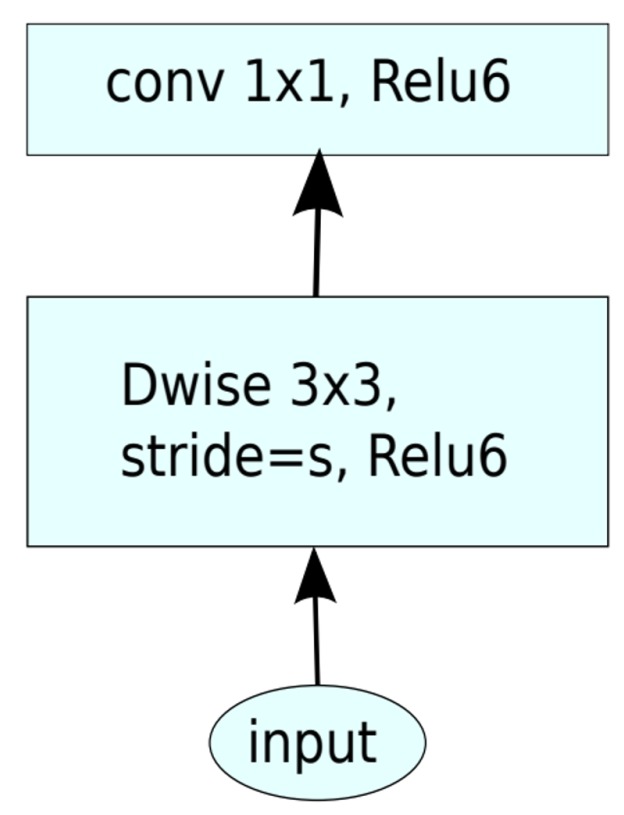
MobileNet [3] uses depth-wise separable convolutions. DWise denotes depth-wise convolution. Skip connections are not used.

The motivation behind MobileNet [3] illustrated in Figure 2 was to reduce the network computational cost by using 3 × 3 depth-wise separable convolutions. Specifically, a depth-wise separable convolution is a form of factorization which reduces computational cost in comparison with a standard convolution. A more comprehensive comparison between a normal convolution and a depth-wise separable convolution is provided in Section 1.2. The study in [3] showed a drawback when evaluated with ImageNet, i.e., the performance of MobileNet decreased by 1%, but the advantage was a substantial reduction in computational cost in comparison with those of the model with a normal 3 × 3 convolution.

**Figure 3:**
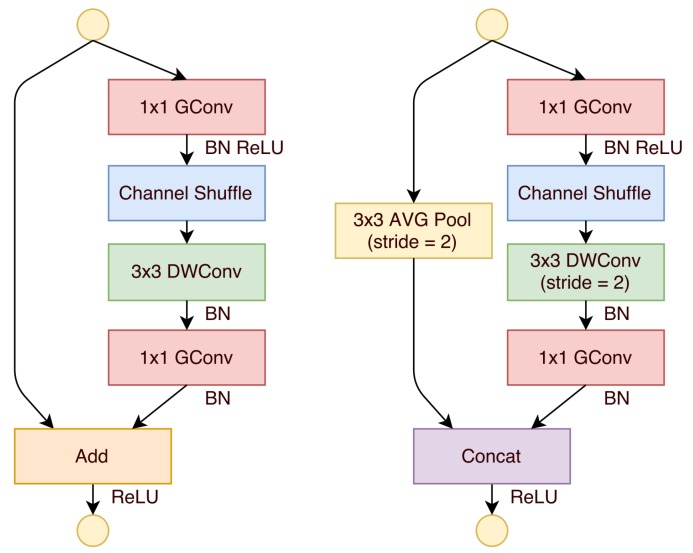
ShuffleNet [4] uses a 3 × 3 convolution for the depth-wise phase of the convolution which is performed after a channel shuffle. DWConv denotes depth-wise convolution. This architecture uses skip connections.

ShuffleNet [4], as illustrated in Figure 3, uses two new operations, i.e., a point-wise group convolution and channel shuffling. A 3 × 3 kernel was used for the depth-wise portion of the depth-wise separable convolution operation to reduce computational cost. The motivation of using a shuffle was to combat pitfalls in group convolutions. Specifically, if multiple group convolutions stack together, output channels are only derived from a small fraction of input channels which impacted performance. Shuffling the channels overcame this problem and led to performance improvements over MobileNet. However, this additional operation, i.e., shuffling, is also a drawback, as it leads to additional computation.

**Figure 4:**
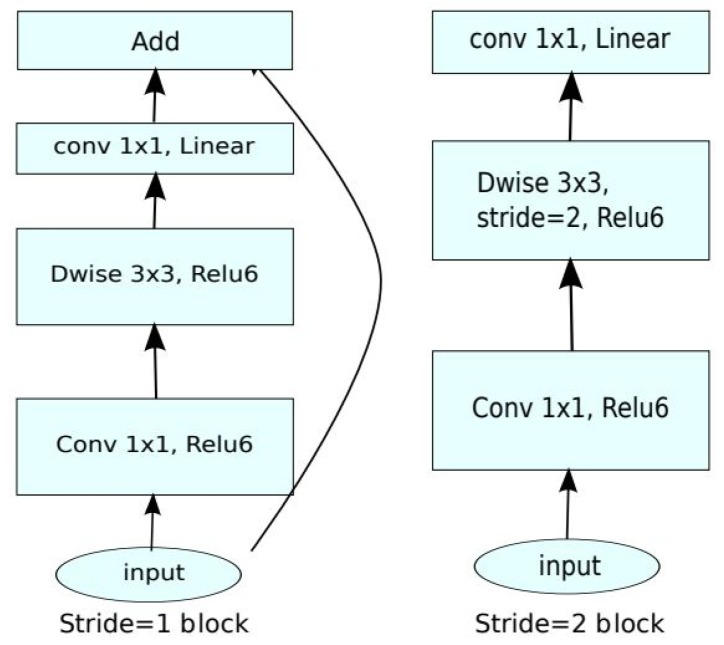
MobileNetV2 [6] uses a 3 × 3 convolution for the depth-wise phase of the convolution and makes use of skip connections. DWise indicates depth-wise convolution.

MobileNetV2 [6], as illustrated in Figure 4, builds on the original MobileNet architecture using 3 × 3 depth-wise separable convolutions but with the addition of an inverted residual structure where shortcut connections are used between thin bottleneck layers to reduce input and output sizes. This model outperformed the state-of-the-art networks such as MobileNet and ShuffleNet, at the time for the evaluation of ImageNet.

**Figure 5:**
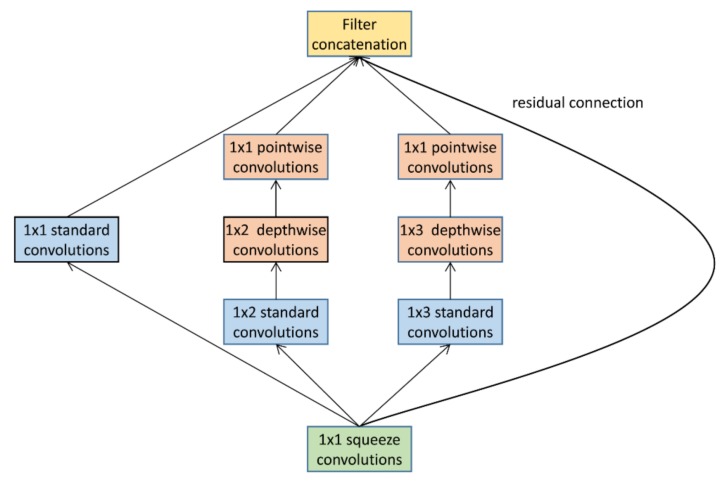
LiteNet [20] takes an inception block and replaces one of the 1x2 convolutions and one of the 1 × 3 convolutions with their depth-wise counterparts, respectively.

LiteNet [20], as illustrated in Figure 5, takes an inception block which contains 1 × 1, 1 × 2 and 1 × 3 standard convolutions arranged side by side and makes modifications (inspired by MobileNet) by replacing half of the 1 × 2 and 1 × 3 standard convolutions with their depth-wise equivalents. Their proposed block, therefore, contains a mix of both standard and depth-wise separable convolutions. Their work also makes use of a SqueezeNet fire block [21] to further reduce the total network parameters. The model was trained on the MIT-BIH electrocardiogram (ECG) arrhythmia database [22] and improved the accuracy rate against baseline models of ≈0.5%. The drawback of their proposed model is the side-by-side structure employed, since side-by-side blocks increase the total number of parameters for a given depth. The inception model originally proposed a side-by-side block to reduce the need to select appropriate filter sizes upfront. By including a variety of different filter sizes side by side, the network could learn which ones are best to use. We have since learnt in related works that the most common filter used is 3 × 3 and deeper models perform better. Therefore, our model eliminates their constraint by focusing on one filter size.

**Figure 6:**
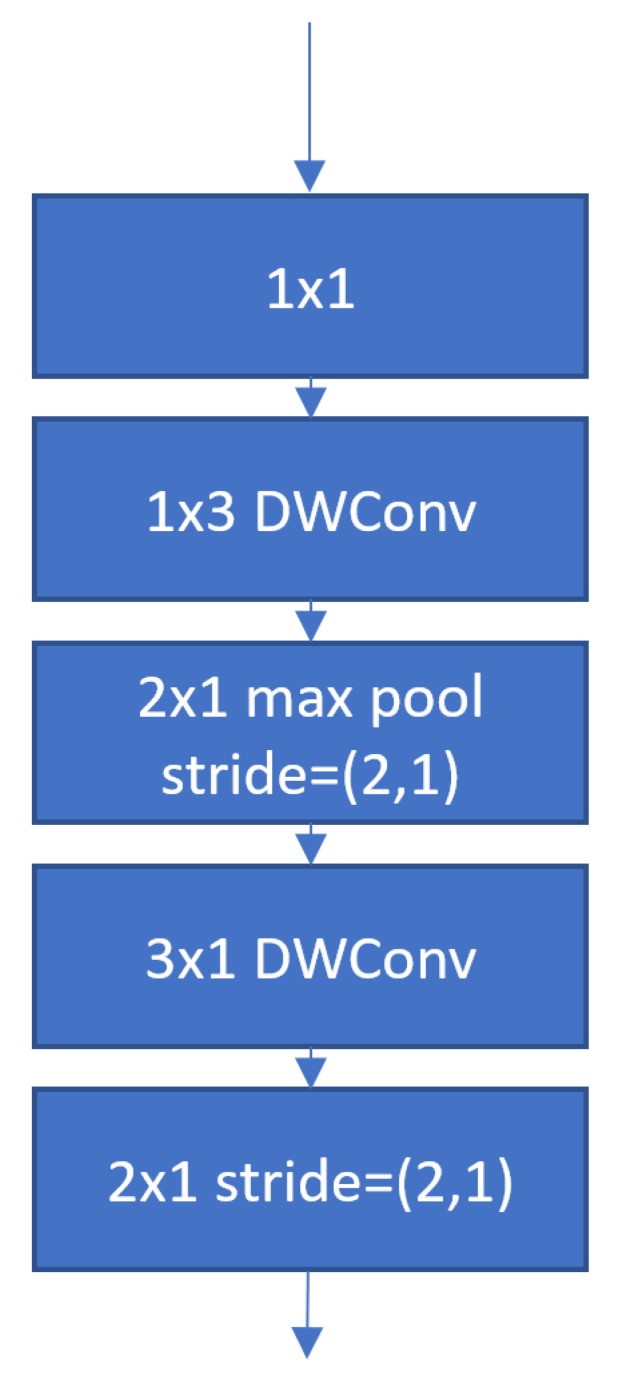
EffNet [5] uses 1 × 3 and 3 × 1 depth-wise separable convolutions to reduce model complexity. DWConv denotes depth-wise convolution.

A common drawback of MobileNet, MobileNetV2 and ShuffleNet is a substantial reduction in the total number of floats-out when downsampling is performed. The authors of EffNet [5] highlighted this as a weakness as the aggressive nature of the reduction is that floats cause a bottleneck which impedes data flow when the model is small, causing them to diverge. The motivation for EffNet as illustrated in Figure 6 was to deploy networks in performance constrained environments and to increase the efficiency of existing off-shelf models. EffNet achieves this by gradually reducing the total number of FLOP outputs throughout the network to avoid bottlenecks. EffNet also replaced 3 × 3 convolutions with pairs of 1 × 3 and 3 × 1 convolutions performed as a depth-wise separable operation to further reduce computational cost. A weakness to such an approach is that the computational saving of performing a 1 × 3 convolution as a depth-wise operation is less than that of a 3 × 3 convolution as elaborated in Section 1.2.

Besides the above methods, post-processing techniques exist which reduce model complexity and therefore the computational cost. Related studies in this field include [23,24,25,26], which employed pruning algorithms for post-processing. These developments indicated that a model can be compressed to reduce complexity, with minimal impact on performance. Ref. [23] is a pruning algorithm based on Taylor series expansion of a cost function which was applied to SqueezeNet [21], resulting in a 67% model reduction. Some limitations of this approach include a 1% drop in accuracy. It obtains better results when training from scratch, rather than using transfer learning on top of a pre-trained network. Ref. [24] prunes based on a filter stability which is calculated during training. As an example, unstable filters are candidates for pruning. This approach was applied to LeNet-5 [27] on MNIST [28], VGG-16 [29] on CIFAR-10 [7], ResNet-50 [13] on ImageNet [30], and Faster R-CNN [31] on COCO [32] and reduced the number of FLOPs by a factor of 6.03X. A limitation to this approach is that it can only be used on new models trained from scratch.

In contrast to post-processing techniques, architecture generation algorithms such as [33,34,35,36,37] have demonstrated that architectures can be automatically generated by exploring different architecture choices and hyper-parameter settings. Ref. [34] used a Q-Learning method [38] with an epsilon-greedy exploration strategy [39] to speed up the time taken when generating new model architectures. The algorithm was able to choose from 1 × 1, 3 × 3 or 7 × 7 convolutions and was trained on CIFAR-10. The approach was able to reduce the time required to generate suitable architectures from 22 days for the current state-of-the-art approach [40] to 3 days with a 0.1% reduction in the error rate. Ref. [33] recently proposed an ageing evolution algorithm which extended the well-established tournament selection in genetic algorithm [41] by introducing an age property to favor younger genotypes. The algorithm chose from 3 × 3, 5 × 5 or 7 × 7 separable convolutions, 1 × 7 then 7 × 1 standard convolutions, 3 × 3 max or average pooling and dilated convolutions. The approach achieved a new state-of-the-art 96.6% top-5 accuracy rate on ImageNet. These evolving model generation methods require additional computational resources owing to the large search space and complex evolving processes with the involvement of fitness evaluations.

Parameter quantization is an area of research which aims to make a network have a smaller memory footprint by compressing 32-bit parameters to 16-bit or even smaller. Related developments such as [42,43,44] have explored compression to various degrees, e.g., including reducing weights to binary values. Bi-Real Net [42] significantly reduced memory footprint and computational cost by setting all weights and activations to binary values. This process was achieved by using a sign function which replaced the true activations and weights with either −1 or 1. It also reduced the memory usage of the previous state-of-the-art 1-bit CNN XNOR-Net [45] by 16 times and reduced computational cost by 19 times. Ref. [44] introduced chunk-based accumulation and floating-point stochastic rounding functions which compressed weights from 32-bit to 8-bit. In comparison with a wide spectrum of popular CNNs, for the evaluation of several benchmark data sets, their network achieved similar accuracy rates as those of the baseline models, but with reduced computational costs. However, the study also indicated that their model suffered from loss of precision over the 32-bit model counterparts.

Learning data augmentation strategies which can be transferred across different data sets and models such as [46] have proved extremely effective at improving model accuracy by discovering novel combinations of data augmentations which can be applied to specific data sets and often transferred to others.

The above studies on pruning algorithms, automatic architecture generation and parameter quantization are examples of related work, which could complement ours and be embedded for future development.

### 1.2. Distinction Between Standard Convolutions and Depth-Wise Separable Convolutions

The following section aims to make a clear distinction between a standard convolution found in our proposed model and depth-wise separable convolutions found in related works, pertaining to their differences in methodology and computational cost. Subscripts have been used because our kernels are not square as they are either 1 × 3 or 3 × 1 in shape.

#### 1.2.1. Standard Convolution

**Figure 7:**
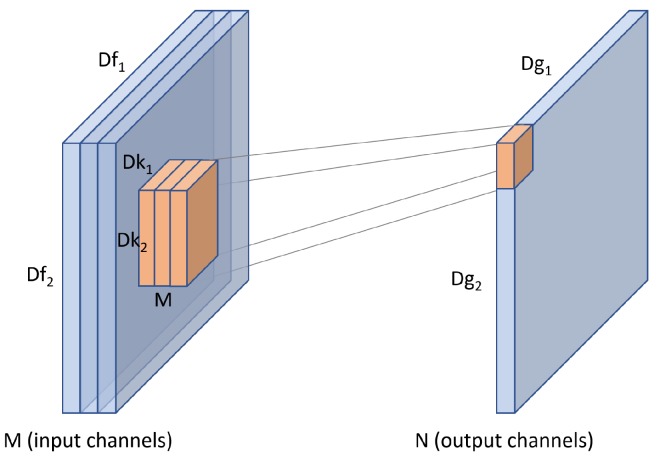
A standard convolution uses a kernel which extends the entire depth of an input.

For an input *f* of size $Df_{1}\times Df_{2}\times M$, a standard convolution uses a kernel *k* which extends the entire depth of the input. The kernel therefore has a size of $Dk_{1}\times Dk_{2}\times M$. Convolving *k* with input *f* produces an output *g* of size $Dg_{1}\times Dg_{2}$ as seen in Figure 7.

We can use *N* such kernels to produce multiple output channels. The computational cost of a standard convolution can be calculated with Equation (1)

(1) $$standard=N\cdot Dg_{1}\cdot Dg_{2}\cdot Dk_{1}\cdot Dk_{2}\cdot M$$

#### 1.2.2. Depth-Wise Separable Convolution

A depth-wise separable convolution is performed in two stages, i.e., a depth-wise stage, and a point-wise stage. To calculate the computational cost of a depth-wise separable convolution, we calculate and sum the computational costs of both phases.

**Figure 8:**
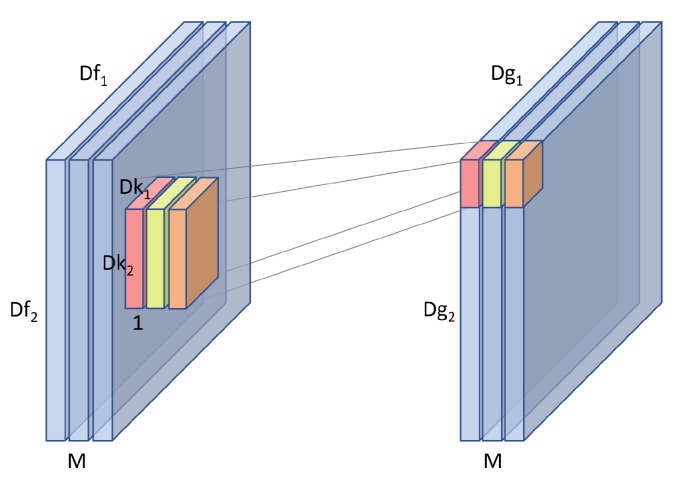
In the depth-wise phase, multiple kernels are used to exploit the entire depth of an input as each kernel only spans one channel.

The depth-wise stage uses a kernel *k* which spans only one channel of input *f*. *M* such kernels are used to span the entire depth of the input to produce an intermediate output *g* of size $Dg_{1}\times Dg_{2}\times M$ as shown in Figure 8.

The computation cost of this phase can be calculated with Equation (2)

(2) $$depthwise=M\cdot Dg_{1}\cdot Dg_{2}\cdot Dk_{1}\cdot Dk_{2}$$

**Figure 9:**
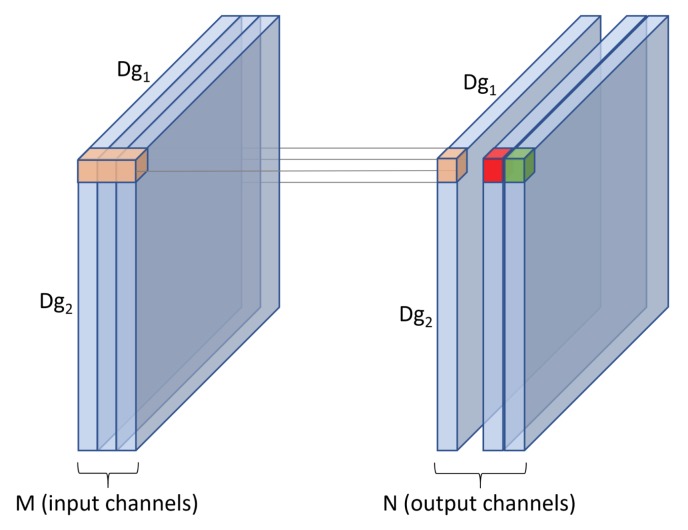
In the point-wise phase, a standard convolution is performed on the intermediate output from the depth-wise phase.

The point-wise stage combines the intermediary output from the depth-wise stage using a standard convolution, commonly with a 1×1 kernel. As with the standard convolution we can have *N* such kernels to produce multiple output channels as shown in Figure 9.

The computational cost of this phase can be calculated with Equation (3)

(3) $$pointwise=N\cdot Dg_{1}\cdot Dg_{2}\cdot M$$

The motivation when using a depth-wise separable convolution is to reduce the computational cost. The cost saving can be calculated as Equation (4) which simplifies to Equation (5).

(4) $$cost=\frac{depthwise+pointwise}{standard}$$

(5) $$cost=\frac{Dk_{1}\cdot Dk_{2}+N}{N\cdot Dk_{1}\cdot Dk_{2}}$$

It is more likely to perform a convolution as a depth-wise convolution rather than a standard convolution when using a 3 × 3 kernel than it is when using a 1 × 3 kernel. This becomes clearer if we compare the savings of both kernel types. Equation (6) shows that a convolution with a 3 × 3 kernel and 64 channels will require only 12.7% of the total FLOPs a normal convolution would require if performed depth-wise, which is a significant saving. On the other hand, Equation (7) illustrates that a convolution with a 1 × 3 kernel and 64 channels will use 34% of the total FLOPs a normal convolution would require. This means that the cost saving is greater on a 3 × 3 kernel, therefore we are more motivated to explore the proposed mechanisms on a 3 × 3 kernel instead of a 1 × 3 kernel.

(6) $$\frac{Dk_{1}\cdot Dk_{2}+N}{N\cdot Dk_{1}\cdot Dk_{2}}=\frac{3\cdot 3+64}{64\cdot 3\cdot 3}=0.127$$

(7) $$\frac{Dk_{1}\cdot Dk_{2}+N}{N\cdot Dk_{1}\cdot Dk_{2}}=\frac{1\cdot 3+64}{64\cdot 1\cdot 3}=0.34$$

## 2. Materials and Methods

Our proposed model consists primarily of groups and blocks. A group is a logical collection of blocks. A group also contains metadata such as to what degree resolution downsampling and widening should be applied. A block is a collection of operations such as convolutions which are performed in a repeatable sequence.

Our model has an initial 3 × 3 convolution, followed by at least one group of blocks. The depth of our model is controlled by increasing or decreasing the number of groups with g∈{1,2,3} and controlling the number of *n* blocks within each group where n≥1. A block consists of batch normalization [47] followed by a pair of 1 × 3 and 3 × 1 standard convolutions and contains a skip connection. A block is defined in Equation (8) from [14] as $x_{l}$ and $x_{l+1}$ represent the input and output of the *l*-th block in the network, respectively. *F* is a residual function and $W_{l}$ is the parameter matrix of the block. Each convolution is preceded with a ReLU [48].

(8) $$x_{l+1}=x_{l}+F\left(x_{l},W_{l}\right)$$

**Figure 10:**
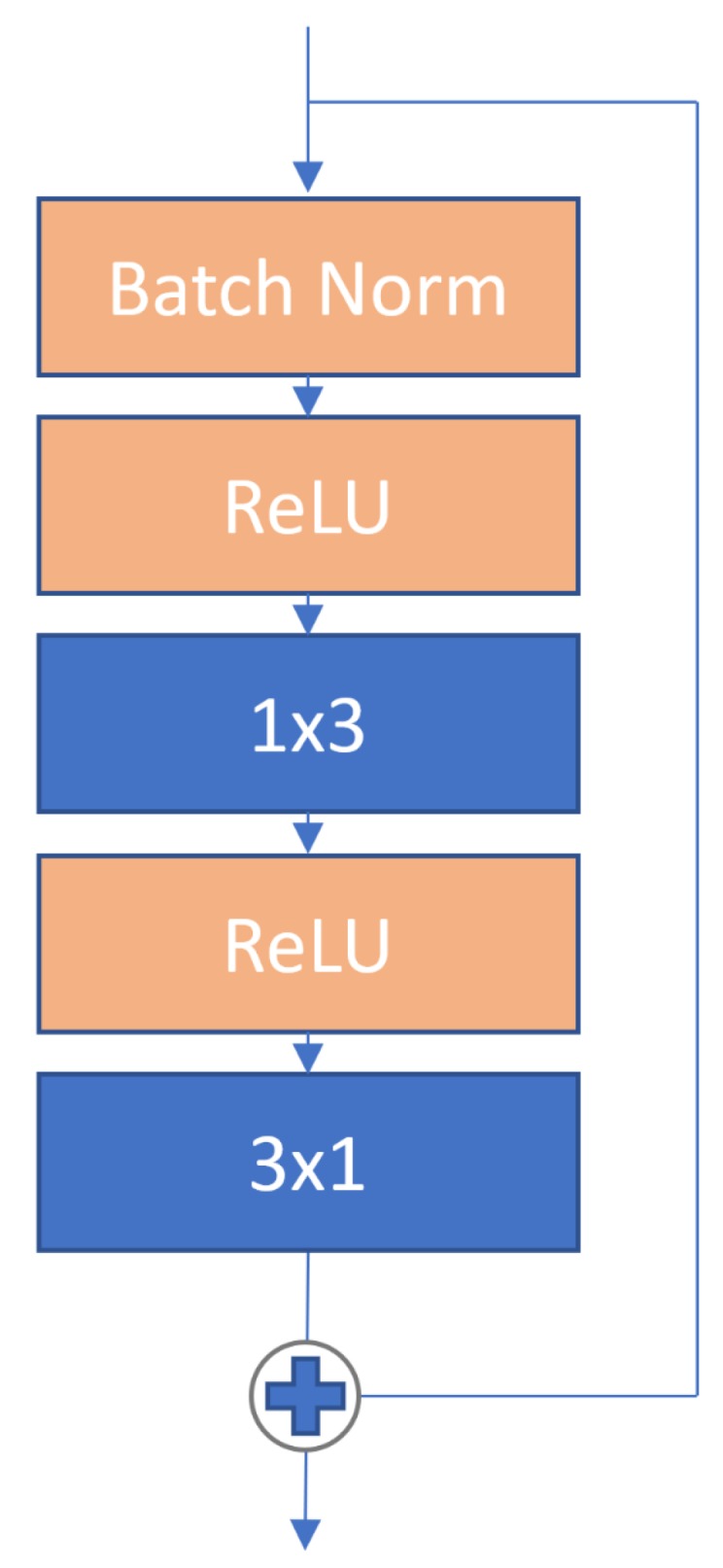
Our network block contains a batch normalization, followed by a pair of 1 × 3 and 3 × 1 standard convolutions. Each convolution is preceded with a ReLU. Each block also contains a skip connection [13].

Our proposed network block is shown in Figure 10.

The width within our model is controlled with a widening factor of *k*. The first block of each group is responsible for increasing width. The initial 3 × 3 convolution has a width of floor(16*k). Group one has a width of floor(16*k), while group two has a width of floor(32*k), and group three has a width of floor(64*k). Except for the initial 3 × 3 convolution, these settings were taken from related studies of [14].

**Figure 11:**
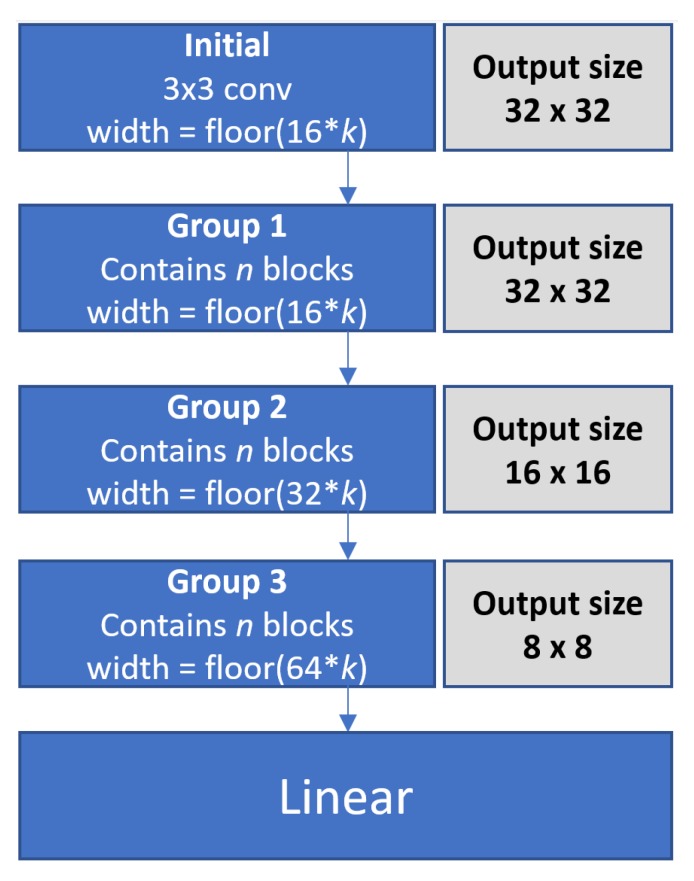
Our network width is controlled by a widening factor *k*. Resolution is reduced within the first blocks of groups two and three if present.

Resolution downsampling is performed in groups two and three if they are present. The first blocks of groups two and three reduce the resolution by performing average pooling using a 2 × 2 filter, which halves the output resolution. Figure 11 shows how depth, width and resolution can be controlled, and how our model is made up of *g* groups, containing *n* blocks.

The linear layer which performs final classification is preceded by batch normalization, ReLU and average pooling.

We opted not to use the data augmentation policies learnt through related works, such as AutoAugment [46], as while they have great potential to improve model performance. We simply opted to instead use mean/std normalization for all data sets so that the architectures themselves can be compared fairly. In addition to this, for the CIFAR-10 data set, we also use the approach taken in [14] of horizontal flip, random crop and padded by 4 pixels. Missing pixels added through padding are repopulated using reflections of the original input image.

### 2.1. Approach to Identify Candidate Models

In this research, we use multiple filtering steps to reduce the architecture search space and identify the optimal network configurations for each test data set. The detailed process is provided below.

Step 1—We calculate the FLOPs for all combinations of groups, i.e., g∈{1,2,3}, number of blocks per group, i.e., n∈{1,2,3,4,5}, data set classes, i.e., c∈{10,43}, and the widening factor in the range of [0.1,2.0] in intervals of 0.01.

Step 2—We filter the results down to networks within a target FLOP range. We set the range to between 50% and 100% of the FLOPs of the smallest baseline benchmark model for each data set. For example, the ShuffleNet large model for CIFAR-10 has the smallest number of FLOPs, i.e., 11.1 million (see Table 2). Therefore, our filtering range for candidate model selection would be between 5.5 and 11.1 million FLOPs.

Step 3—We narrow the results down further by selecting the minimum and maximum widths for every unique combination of *g* and *n*. This process results in a list of configurations which contain two candidate models for each unique combination of *g* and *n*.

Step 4—We train the narrowed list of model configurations obtained from Step 3 using a reduced epoch count of 25 to reduce training cost. Then the trained models are tested with the test data set.

Step 5—We finally select the most promising models (e.g., 2–3 models) based on the test accuracy rates obtained in Step 4 as candidate models for full training with 200 epochs.

Automatic architecture generation techniques, such as Particle Swarm Optimization-based deep CNN model generation, will also be explored in future directions.

### 2.2. Complexity Analysis

The main indicator of computational cost, used in efficiency-focused related studies, such as our benchmark models MobileNet [3], MobileNetV2 [6], ShuffleNet [4] and EffNet [5], is to report the number of floating-point operations, i.e., FLOPs. Therefore, we adopt the same indicator for direct computational cost comparison.

Influential studies such as [3] highlighted that computational cost depends multiplicatively and therefore varies based on the number of FLOPs. The number of FLOPs for a standard convolution as used in this study depends on the number of input channels *M*, the number of output channels *N*, the kernel size $Dk_{1}\cdot Dk_{2}$ and the feature map size $Df_{1}\cdot Df_{2}$, as shown in Figure 7. Full details of how convolutional cost is calculated in terms of FLOPs, and a complexity analysis of the proposed modifications and customizations, i.e., cost differences between standard and depth-wise convolutions, and differences between 3 × 3 and 1 × 3 convolutions, are provided in Section 1.2. The impact of selecting different widening factors *W* to the computational cost of a convolution within our model can be compared by scaling Equation (1) from [3] with *W*, as illustrated in Equation (9). This shows that for the proposed models which share the same numbers of groups and blocks, the computational cost of convolutions scales proportionately with *W*. In other words, when other network configurations remain intact, the computational cost increases as the widening factor scales up and vice versa.

(9) $$cost=N\cdot Dg_{1}\cdot Dg_{2}\cdot Dk_{1}\cdot Dk_{2}\cdot M\cdot W$$

## 3. Results

We present our experimental studies in this section. Specifically, in Section 3.1, we provide a detailed overview of the test data sets employed in our experiments, as well as the model training scheme. We compare our approach against efficiency-focused benchmark models and the 3 × 3 standard convolution-based models in Section 3.2 and Section 3.3, respectively. We conduct in-depth model and result analysis in Section 3.4.

### 3.1. Data Sets

The data sets used within our experiments include CIFAR-10 [7], SVHN [8] and GTSRB [9]. These data sets offer realistic and varied representations of the types of image classification problems that could be encountered in resource-constrained environments. All these employed data sets have pre-defined training and test sets meaning that all benchmark models and our model have been trained and tested under the same experimental settings, i.e., using the same data splits, samples and image resolutions. The selected data sets are summarized in Table 3 and introduced in more detail in the following subsections.

#### 3.1.1. CIFAR-10

**Figure 12:**
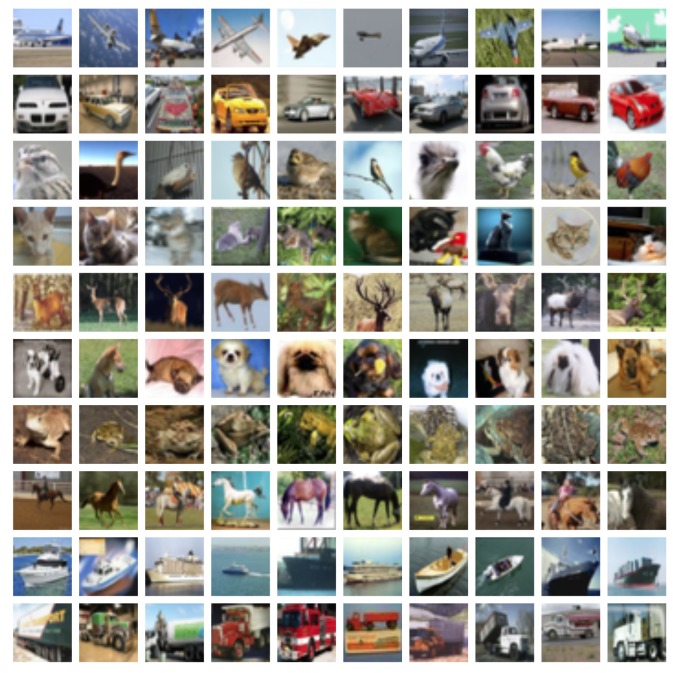
Example images extracted from the CIFAR-10 data set.

The CIFAR-10 data set [7] consists of 60,000 images in 32 × 32 resolutions. They are split into 50,000 and 10,000 samples for training and test, respectively. Each image is categorized as one of the ten classes, including airplane, automobile, bird, cat, deer, dog, frog, horse, ship and truck. Figure 12 illustrates some example images extracted from this data set.

#### 3.1.2. SVHN

**Figure 13:**
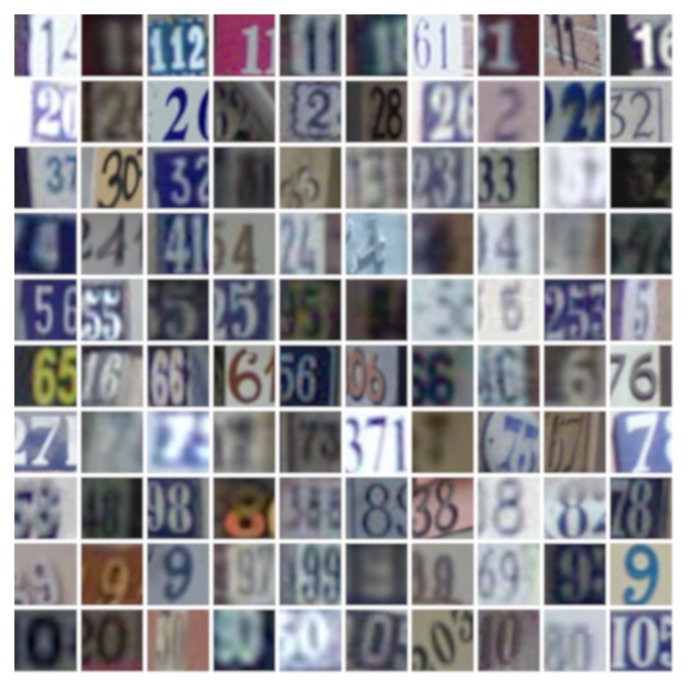
Example images extracted from the SVHN data set.

The SVHN data set [8] contains house numbers obtained from Google Street View images. It is divided into 73,257 training and 26,032 test images. We used a 32 × 32 crop of the original images which produces a MNIST-like data set. In this format, each image contains one digit of interest belonging to one of 10 classes, i.e., a number between 0–9, along with some distracting digits in both sides of each image. Some example images in the SVHN data set are shown in Figure 13.

#### 3.1.3. GTSRB

**Figure 14:**
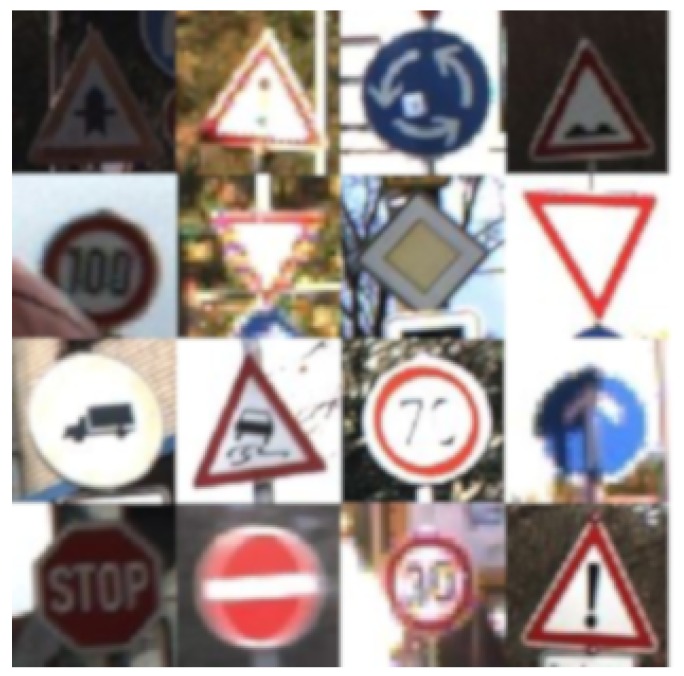
Example images extracted from the GTSRB data set.

The GTSRB [9] data set is composed of 51,839 images of traffic signs covering 43 different classes of signs including stop, no entry and speed limits. Images within the same class are of different physical signs with various lighting conditions and image qualities. The data set is split into 39,209 and 12,630 samples for training and test, respectively. Figure 14 illustrates some example images from this data set.

**Figure 15:**
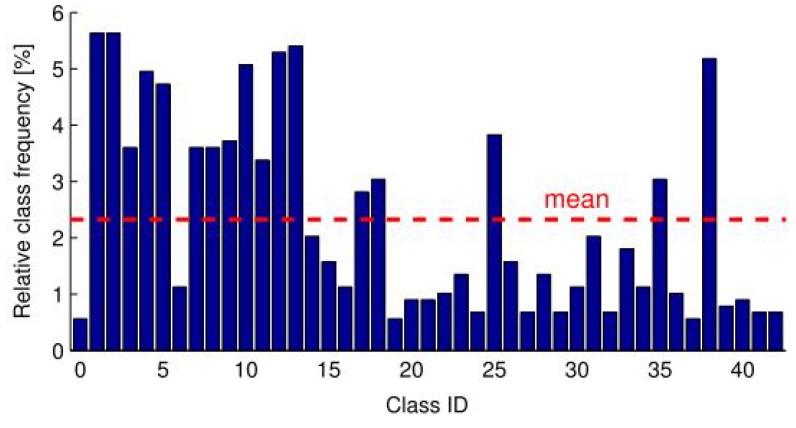
The imbalanced class distributions within the GTSRB data set.

Moreover, GTSRB illustrates imbalanced class distributions with relatively small numbers of examples for some of the classes as shown in Figure 15. Such characteristics make the data set challenging as it is prone to overfitting.

#### 3.1.4. Training Scheme

When training using the CIFAR-10 [7] and GTSRB [9] data sets, we adopt stochastic gradient descent (SGD) optimizer with cross-entropy loss. A total of 200 epochs are used for model training. The initial learning rate lr is set to 0.1, and dropped by lr*0.2 at epochs 60, 120 and 160. For the SVHN [8] data set, we use the Adam optimizer [49] with cross-entropy loss. A total of 200 epochs with a fixed learning rate of 0.001 have been applied. The above experimental settings are obtained by trial-and-error to achieve the best model performance. We employ the mean result for a total of 5 runs as the main criterion for comparison. The proposed model has been implemented using PyTorch [50].

### 3.2. Comparison Against Efficiency-Focused Benchmark Models

A comprehensive evaluation has been conducted to compare the proposed block architectures against the baseline efficiency-focused models, i.e., EffNet [5], MobileNet [3], MobileNetV2 [6] and ShuffleNet [4]. Our evaluation also compares the proposed approach directly against a standard 3 × 3 convolution method typically used in state-of-the-art accuracy-focused models. We denote the depth settings for our proposed model as IoTNet-*g*-*n* where *g* is the number of groups, and *n* is the number of blocks within each group. IoTNet-3-2 for example contains three groups, each of which contain two blocks. We adopt the same experimental settings to ensure a fair comparison, i.e., by using the aforementioned data sets with pre-defined training and test sets and input resolutions. The experimental results are presented separately for each test data set.

The evaluation results of the baseline networks were obtained from the work of EffNet [5]. The authors of EffNet constructed models of comparable sizes by adjusting the networks width using a widening factor, adding additional layers or a combination of both. A summary of the models with brief configuration descriptions employed for performance comparison in this research is provided in Table 4. Further details can be obtained from their original studies [5].

#### 3.2.1. Evaluation Using CIFAR-10

The baseline models are split into two categories according to model sizes, measured in FLOPs. We adopt the multi-filtering steps as discussed in Section 2.1 to identify suitable candidate models for each network category, which contain fewer FLOPs than those of the baselines. Three candidate models of the proposed approach were identified and trained with 200 epochs on CIFAR-10 for both large and small network configurations, respectively. The detailed results are shown in Table 5.

As illustrated in Table 5, all top candidate models of the proposed approach contain three groups since those with two groups performed worse than such networks, while those with one group performed worse than the models with two groups. This indicates that multiple downsampling stages and network depth are important factors. Candidate models favored a balance between depth and the widening factor owing to the comparatively challenging nature of CIFAR-10. The empirical results also indicate that shallow but wide models, or deep and narrow models performed worse.

Table 2 shows the detailed results of the best candidate models and the baseline networks for the CIFAR-10 data set while Table 6 indicates the performance improvements of the best performing candidate models over the benchmark networks. The results in both tables are split into two categories according to model sizes.

Within the first groups (i.e., the larger networks) of Table 2 and Table 6, we compare our best candidate model against larger versions of the efficiency-focused benchmark networks. The best performing benchmark baseline model is EffNet V1 large with an accuracy rate of 85.02%, with 79.8 million FLOPs. Our proposed model achieves an accuracy rate of 89.9% with 9.9 million FLOPs. It outperforms EffNet V1 large by 4.88% in terms of accuracy with 87.59% fewer FLOPs. The proposed model also delivers a considerable improvement in terms of accuracy compared to MobileNetV2 with a 13.43% accuracy improvement, with 39.63% fewer FLOPs.

Within the second group (i.e., the networks with smaller configurations), we compare our proposed model against smaller versions of the efficiency-focused benchmark models. The best performing baseline model is MobileNet with an accuracy rate of 77.48% and 5.8 million FLOPs. Our proposed model achieves an accuracy rate of 87.19% with 4.2 million FLOPs, i.e., an improvement of 9.71%, with 27.59% fewer FLOPs against those of MobileNet. The proposed model also beats ShuffleNet with a 9.89% accuracy improvement, with 10.64% fewer FLOPs.

The results also indicate that the difference between MobileNet and MobileNet large, and the difference between ShuffleNet and ShuffleNet large, in terms of performance when scaling up from smaller to larger sizes, result in less than a 1% improvement in accuracy, respectively. On the contrary, the performance improvement of the proposed models, i.e., between IoTNet-3-4 and IoTNet-3-2, is by 2.71% indicating that diminishing returns when scaling up MobileNet and ShuffleNet, which the proposed model overcomes.

#### 3.2.2. Evaluation Using SVHN

We adopt a multi-filtering search strategy to identify suitable candidate models which contain fewer FLOPs than those of the baselines. Two top candidate models were subsequently identified and trained with 200 epochs on SVHN. The evaluation results on the test set are shown in Table 7.

As indicated in Table 7, the candidate models containing three groups performed the best which again indicates that multiple downsampling stages and depth are influential factors. Owing to the less challenging nature of SVHN, the candidate models favored depth over width. Since SVHN contains digits which vary much less between samples than a more general data set would such as CIFAR-10, this indicates that fewer filters are required to extract fine detail and perform classification well. Therefore, less width was required.

Within Table 8 and Table 9, we compare the best candidate model against efficiency-focused benchmark models on the SVHN data set. Motivated by the related research [5] where the comparison was conducted using one category of smaller networks owing to the simplicity of the problem, we perform the comparison for SVHN using a similar style, i.e., purely with the smaller network category. As illustrated in Table 8 and Table 9, the best performing benchmark baseline model is EffNet V1 with an accuracy rate of 88.51%, with 517.6 kFLOPs. Our proposed model achieves an accuracy rate of 89.22%, with 499.7 kFLOPs, and outperforms EffNet V1 by 0.71% in terms of accuracy with 3.46% fewer FLOPs. It also makes a considerable improvement, i.e., 6.49%, in terms of accuracy with 31.84% fewer FLOPs as compared with those of ShuffleNet. Our model is also able to make a significant reduction, i.e., 57.03% in FLOPs, when compared against MobileNetV2 while also improving accuracy by 2.51%. The empirical results indicate that the proposed architecture substantially reduces the trading of accuracy over computational cost by making significant reductions in FLOPs while improving performance across the board.

#### 3.2.3. Evaluation Using GTSRB

The baseline models are split into two categories according to model sizes, measured in FLOPs. We adopt a multi-filtering search method to identify suitable candidate models with fewer FLOPs than those of the baselines, for each network category. Three candidate models were identified for each network configuration, which were subsequently trained with 200 epochs and tested on GTSRB. The detailed results are shown in Table 10.

As shown in Table 10, all top candidate models contained three groups. They outperformed all the networks with one group or two groups, which ascertains the importance of multiple downsampling stages and network depth. The candidate models required less width than on those used for CIFAR-10, yet more width than those tested upon SVHN. Since GTSRB is imbalanced with comparatively more classes (i.e., 43) and contains images with a range of lighting conditions, it is more challenging than SVHN. Therefore, more filters are required in the models than those used in SVHN. On the other hand, GTSRB consists of images with road traffic signs which are comparatively consistent in design and have fewer variations. Thus, it is less challenging than CIFAR-10. Therefore, fewer filters are required than those used in CIFAR-10. Also, the results indicate that shallow but wide, or deep and narrow models performed worse. As an example, the empirical results in Table 10 indicate that when constructing small models, depth must be compromised with width. This can be observed by the improvement in performance when reducing the number of blocks per group from three to two, while increasing width.

Within Table 11 and Table 12, we compare the best candidate model for each network category against efficiency-focused benchmark models on the imbalanced GTSRB data set. For the larger network comparison, the best performing benchmark baseline model is MobileNetV2 with an accuracy rate of 90.74%, with 710.7 kFLOPs. The proposed model achieves an accuracy rate of 93.17%, with 531.0 kFLOPs. It outperforms MobileNetV2 by 2.43% in terms of accuracy with 25.28% fewer FLOPs. It also makes a significant improvement, i.e., 5.02%, in terms of accuracy with 0.38% fewer FLOPs as compared with those of MobileNet. Our model is also able to make a significant reduction in FLOPs, i.e., 24.63%, when compared against EffNet V2 while also improving accuracy by 2.77%. The empirical results also indicate that when scaling down to 301.6 kFLOPS, it was not possible to increase the width beyond 0.18 but at the same time also staying within the target FLOP range of below 344.1 kFLOPs. Therefore, further studies will be conducted around different widening schemes to address this.

### 3.3. Evaluation Against 3 × 3 Standard Convolutions

To prove the effectiveness of our proposed architecture against a scaled-down state-of-the-art model based on the popular 3 × 3 standard convolution, we construct a 3 × 3-based model by replacing our 1 × 3 and 3 × 1 pairs with a 3 × 3 convolution for comparison. We then scale our proposed model and its 3 × 3 standard convolution counterpart to contain between 1 to 10 million FLOPs. The 3 × 3 configuration closely resembles scaled-down variants of popular models proposed by [13,14]. With some minor alterations to the width calculation, it would also resemble the architecture proposed by [16]. We train both models on CIFAR-10, SVHN and GTSRB data sets as discussed earlier.

#### 3.3.1. Evaluation against 3 × 3 Standard Convolution-Based Models Using CIFAR-10

**Figure 16:**
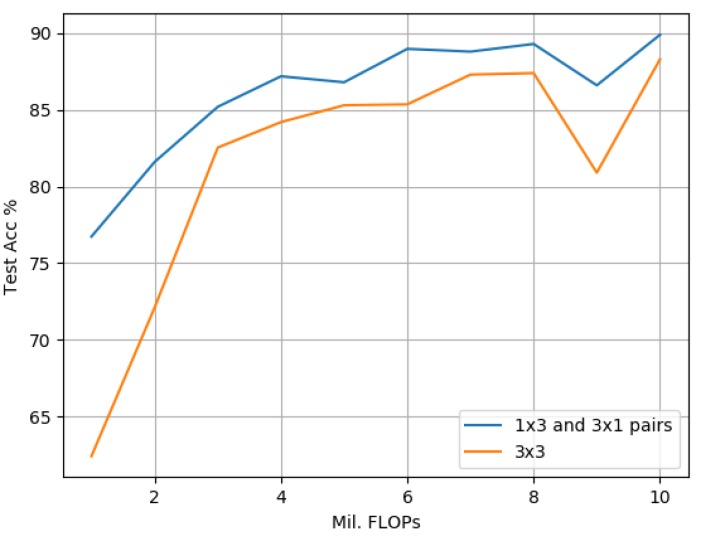
CIFAR-10: The proposed model based on 1 × 3 and 3 × 1 convolution pairs compared with a 3 × 3-based approach. Both variants are scaled to match in terms of FLOPs ranging from 1 to 10 million.

Figure 16 demonstrates that on the CIFAR-10 data set, our proposed model offers significantly improved accuracy rates over its 3 × 3 counterpart in all cases when scaled between 1 and 10 million FLOPs. The empirical results also indicate that scaling both model variants to sizes greater than 3 million FLOPs results in an improvement of accuracy, but with greater diminishing returns between the model complexity and the observed accuracy improvement.

#### 3.3.2. Evaluation against 3 × 3 Standard Convolution-Based Models Using SVHN

**Figure 17:**
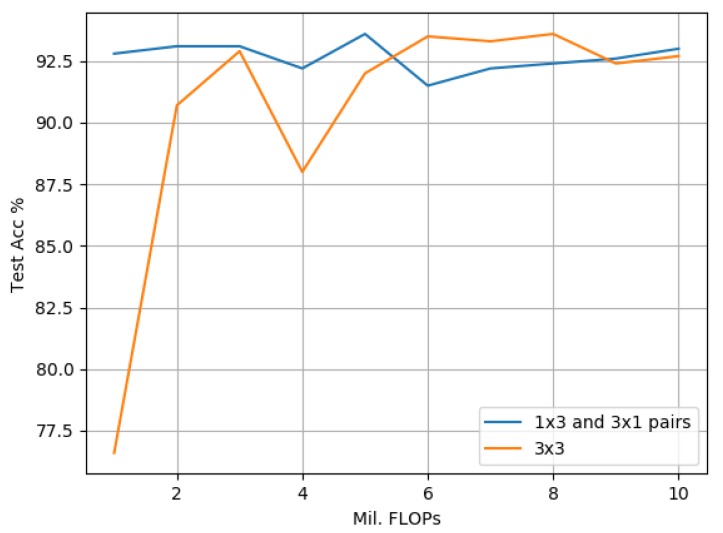
SVHN: The proposed model based on 1 × 3 and 3 × 1 convolution pairs compared with a 3 × 3-based approach. Both variants are scaled to match in terms of FLOPs ranging from 1 to 10 million.

Figure 17 compares our proposed model against its 3 × 3 counterpart on the SVHN dataset. The empirical results indicate that due to SVHN representing a simpler problem when compared to CIFAR-10, scaling the 3 × 3 model to more than 3 million FLOPs does not result in any significant performance improvements. Also, the accuracy rate achieved by the 3 × 3 model when scaled to 3 million FLOPs is surpassed by that of the proposed model containing just 1 million FLOPs. This indicates a significant reduction in computational cost by using the proposed model. For experiments scaled above 6 million FLOPs, the proposed model achieves greater accuracy when using just 5 million FLOPs, which represents another significant reduction in computational cost.

#### 3.3.3. Evaluation against 3 × 3 Standard Convolution-Based Models Using GTSRB

**Figure 18:**
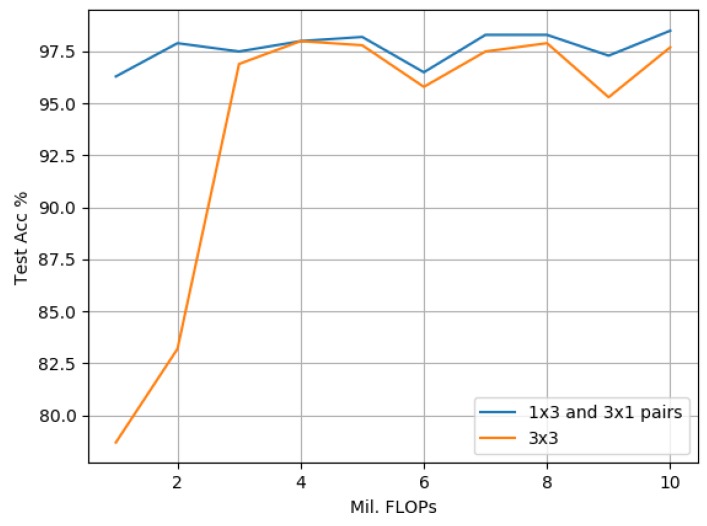
GTSRB: The proposed model based on 1 × 3 and 3 × 1 convolution pairs compared with a 3 × 3-based approach. Both variants are scaled to match in terms of FLOPs ranging from 1 to 10 million.

Figure 18 compares the proposed model against its 3 × 3 counterpart on the GTSRB data set. The empirical results indicate that scaling either model larger than 3 million FLOPs does not result in any significant real-world accuracy gains. The empirical results for models containing between 1 and 3 million FLOPs indicate significant accuracy improvements with the proposed model outperforming its 3 × 3 counterpart throughout. While both models with 4 million FLOPs result in the same accuracy rates, the proposed model scaled above 4 million FLOPs again shows superior performance over its 3 × 3 counterpart.

#### 3.3.4. Computational Comparison

We evaluate our model’s suitability for deployment on a resource-constrained environment by measuring the time and space required to process one image from a batch size of 128 images with 32 × 32 resolutions. The tests are performed using our best performing models obtained from Table 2, Table 8 and Table 11. We measure time and space using a Raspberry Pi 3 Model B+ device and compare them against those of a desktop PC. The specifications of both devices can be found in Table 13, while the results of the tests are recorded in Table 14. All tests are performed using the CPUs, as resource-constrained environments are often lack of GPUs. We generate the processing time per image for both environments as follows. First, the time spent for the processing of a batch of 128 images in milliseconds is collected, then we divide it by 128 to obtain a mean time elapsed per image.

As expected, the processing time of the Raspberry Pi is longer than that of the desktop PC owing to the significantly faster CPU in the PC. However, the empirical results indicate that the time required to process one image on the Raspberry Pi is very reasonable, ranging between 4.06 ms on our smallest model and 87.5 ms on our largest model. The empirical results also indicate that indeed time is correlated with FLOPs as our slowest model was also the largest in FLOPs. The space requirements across all data sets were well within the bounds of both devices meaning that multiple models could comfortably be deployed within both environments simultaneously. Space could be further reduced if required by decreasing the batch size from 128 to a suitable lower value.

### 3.4. Discussion

The related studies such as [3,4,6] employ depth-wise separable convolutions as a strategy to reduce computational cost, which have proven to be successful pertaining to cost when compared with much larger, state-of-the-art standard convolution-based models such as ResNet. However, in comparison with the most recent works such as [5,20], they are still quite large, e.g., the MobileNet models contain between 41 and 569 million FLOPs. For much smaller models, more suited to constrained IoT devices, we find that our approach of trading accuracy with computational cost by factorizing 3 × 3 standard convolutions into pairs of 1 × 3 and 3 × 1 standard convolutions leads to significant improvements over the efficiency-focused benchmark models. The empirical results indicate that depth-wise separable convolution-based networks scale down worse than our approach due to a lack of parameters at smaller scales, as demonstrated in our experimental studies. This detrimentally impacts their model training processes as well as their performance as indicated within Table 2, Table 8 and Table 11.

Comparing our proposed approach against scaled-down 3 × 3 standard convolution-based models as illustrated in Figure 16, Figure 17 and Figure 18, the empirical results indicate that on all data sets, the proposed model outperforms its 3 × 3 counterpart greater at smaller scales, i.e., with less than 3 million FLOPs. One explanation for this was provided by [13], which highlighted that the important factors to overall model accuracy are network depth and multiple downsampling stages. In other words, deep models that perform downsampling in multiple stages are likely to lead to promising accuracy rates, while shallower models with fewer downsampling operations are more inclined to suffer from poor performance. This is confirmed by our findings in Table 5, Table 7 and Table 10 where our best proposed candidate models always contained three groups. A second explanation pertaining to an interesting side effect to our approach of factorizing a 3 × 3 standard convolution into a pair of 1 × 3 and 3 × 1 convolutions is that it also doubles model depth. The empirical results indicate that this increase in depth was a key factor leading to the significant performance improvements observed in our studies. An increase in depth can, however, lead to overfitting within larger models, i.e., over 3 million FLOPs, which is indicated by a narrower improvement in performance at larger scales against its 3 × 3 counterparts. As the proposed model is designed to improve performance at smaller scales for resource-constrained environments, we argue that this trade-off is acceptable and could be addressed in future directions by incorporating with cutting-edge data augmentation strategies such as [46].

## 4. Conclusions

In this research, we have proposed a new deep architecture, i.e., IoTNet. Based on pairs of 1 × 3 and 3 × 1 standard convolutions, the empirical results confirm that the proposed architecture greatly improves the trade-off between accuracy and computational cost over existing, depth-wise-based approaches which are typically used in efficiency-focused models.

We report state-of-the-art accuracy improvement over influential efficiency-focused architectures such as MobileNetV2 on CIFAR-10 of 13.43% with 39.63% fewer FLOPs, over ShuffleNet on SVHN of 6.49% with 31.84% fewer FLOPs and over MobileNet on GTSRB of 5% with 0.38% fewer FLOPs. We also outperform the current state-of-the-art efficiency-focused model, EffNet (EffNet V1 or EffNet V2), across all data sets, i.e., improving accuracy on CIFAR-10 by 9.7% with 13% fewer FLOPs, on SVHN by 1.92% with 58.5% fewer FLOPs and on GTSRB by 2.77% with 24.63% fewer FLOPs.

Moreover, the experimental studies also indicate that the proposed model delivers greater accuracy with a lower computational cost in comparison with those of the scaled-down 3 × 3 convolution-based counterpart model, representative of state-of-the-art WideResnet, ResNet and PyramidNet.

The benefit of our work is that more powerful models can now be deployed within tightly constrained environments. We believe this is significant as the use cases for CNNs within resource-constrained environments are extremely broad, e.g., IoT and smartphone-based deployments, medical diagnosis, image and video analysis. Lighter and faster models also enable researchers to prototype ideas faster with fewer resources.

Extensions of our work may include using augmentation strategies highlighted in related works such as [46] to further improve performance. Methods such as these will be especially helpful on imbalanced data sets such as GTSRB. We also aim to explore hyper-parameter fine-tuning [51,52,53] using automated processes and parameter quantization techniques to further reduce the memory footprint of the proposed architecture.

## Figures and Tables

**Table 1:** Comparative analysis of related studies.

Model	Kernel	Convolution Type	Emphasis	Methodologies and Strengths	Drawbacks
AlexNet [15]	mixed	standard	accuracy	Demonstrated how the model depth was essential for performance	Contained large kernels which are less efficient. Outperformed by subsequent studies
ResNet [13]	3 × 3	standard	accuracy	Used skip connections to enable training deeper networks	A slim but deep state-of-the-art model, not designed for constrained environments
Inception [17,18]	mixed	standard	accuracy	Trained deeper networks using sparsely connected network architectures, i.e., by using a variety of kernel sizes side by side	The employed side-by-side model increased model complexity
WideResnet [14]	3 × 3	standard	accuracy	Demonstrated that widening a residual network can decrease its depth and improve its performance	A state-of-the-art model, not designed for constrained environments. Less efficient at smaller scales than our approach
PyramidNet [16]	3 × 3	standard	accuracy	Gradually increasing the feature map size of deep networks led to performance improvements on ResNet	A deep state-of-the-art model, not designed for constrained environments. Gradual depth increase led to a larger model size
MobileNet [3,6]	3 × 3	depth-wise	efficiency	Traded accuracy with efficiency by using depth-wise separable convolutions	Contained bottlenecks during downsampling which impeded data flow
ShuffleNet [4]	3 × 3	depth-wise	efficiency	Shuffling channels helped information flowing when performing depth-wise separable convolutions	Shuffle resulted in additional operations and contained bottlenecks which impeded data flow
EffNet [5]	1 × 3 and 3 × 1	depth-wise	efficiency	Factorized 3 × 3 depth-wise convolutions into 1 × 3 and 3 × 1 depth-wise convolutions to reduce complexity. Addressed bottlenecks of prior efficiency-focused models	Based on depth-wise separable convolutions which traded accuracy with efficiency less optimally than our approach
LiteNet [20]	1 × 2 and 1 × 3	depth-wise and standard	efficiency	Combined ideas from Inception and MobileNet	A combination of drawbacks of Inception and MobileNet (see above)
Ours	1 × 3 and 3 × 1	standard	efficiency	Factorized 3 × 3 into 1 × 3 and 3 × 1 standard convolutions to retain the strength of standard convolutions, i.e., superior performance while reducing model complexity	Designed for constrained environments and not to outperform state-of-the-art accuracy-focused models in extremely large configurations on GPU machines.

**Table 2:** Evaluation results for the CIFAR-10 data set grouped by network sizes in FLOPs. The first group contains larger configurations, while the second group comprises smaller ones.

Model	Widening Factor *k*	Mean Acc	Mil. FLOPs
EffNet V1 large	0.99	85.02%	79.8
MobileNet large	0.14	78.18%	11.6
ShuffleNet large	0.14	77.90%	11.1
EffNet V1	0.14	80.20%	11.4
EffNet V2	0.22	81.67%	18.1
MobileNetV2	0.20	76.47%	16.4
**IoTNet-3-4**	**0.7**	**89.9%**	**9.9**
MobileNet	0.07	77.48%	5.8
ShuffleNet	0.06	77.3%	4.7
**IoTNet-3-2**	**0.68**	**87.19%**	**4.2**

**Table 3:** Overview of the data sets used in our experiments.

Data Set	Total Sample Size	Training Samples	Test Samples	Image Resolution
CIFAR-10	60,000	50,000	10,000	32 × 32
SVHN	99,289	73,257	26,032	32 × 32
GTSRB	51,839	39,209	12,630	32 × 32

**Table 4:** A summary of models used for evaluation. We have referenced two variations of EffNet introduced by [5] as EffNet V1 and EffNet V2.

Model Name	Brief Description
IoTNet-*g*-*n*	The proposed model with *g* as the number of groups, and *n* as the number of blocks per group
EffNet V1	An implementation of EffNet [5]. Model architecture contains 1 × 3 and 3 × 1 depth-wise separable convolution and pooling-based blocks
EffNet V1 large	As per EffNet V1 with two additional layers and more channels
EffNet V2	As per EffNet V1, introduced also in [5] in response to MobileNetV2, model contains minor changes relating to network expansion, extension rates (depth and width) and the replacement of ReLU on the point-wise layers with leaky ReLU
MobileNet	An implementation of MobileNet [3] of varying widths. Model architecture contains 3 × 3 depth-wise separable convolutions
MobileNet large	As per MobileNet implementation with two extra layers
MobileNetV2	An implementation of MobileNetV2 [6] of varying widths. Model contains 3 × 3 depth-wise convolutions and inverted residual structures where shortcut connections are between bottleneck layers
ShuffleNet	An implementation of ShuffleNet [4] of varying widths. Model contains 3 × 3 depth-wise convolutions in addition to point-wise group convolution and channel shuffle
ShuffleNet large	As per ShuffleNet implementation with two extra layers

**Table 5:** Accuracy of the best candidate models found using multi-filtering search, then trained and tested on CIFAR-10. The first group contains networks with larger configurations, while the second group comprises smaller ones.

Model	Widening Factor *k*	Mean Acc	Mil. FLOPs
IoTNet-3-2	1.08	89.79%	11
**IoTNet-3-4**	**0.7**	**89.9%**	**9.9**
IoTNet-3-3	0.66	88.98%	6.2
**IoTNet-3-2**	**0.68**	**87.19%**	**4.2**
IoTNet-3-2	0.5	81.47%	2.6
IoTNet-3-3	0.41	83.49%	2.5

**Table 6:** The improvements of the proposed best model for the CIFAR-10 data set over the state-of-the-art networks, grouped by network sizes.

Model	Acc Improvement	FLOPs Saving
EffNet V1 large	4.88%	**87.59%**
MobileNet large	11.72%	14.66%
ShuffleNet large	12.0%	10.81%
EffNet V1	9.7%	13.16%
EffNet V2	8.23%	45.3%
MobileNetV2	**13.43%**	39.63%
MobileNet	9.71%	**27.59%**
ShuffleNet	**9.89%**	10.64%

**Table 7:** Accuracy of the best candidate models found using the multi-filtering search, then trained and tested on SVHN.

Model	Widening Factor *k*	Mean Acc	kFLOPs
**IoTNet-3-5**	**0.14**	**89.22%**	**499.7**
IoTNet-3-2	0.21	88.4%	474.3

**Table 8:** Evaluation results for the SVHN data set.

Model	Widening Factor *k*	Mean Acc	kFLOPs
EffNet V2	0.34	87.3%	1204.2
MobileNetV2	0.33	86.71%	1162.8
EffNet V1	0.14	88.51%	517.6
MobileNet	0.22	85.64%	773.4
ShuffleNet	0.21	82.73%	733.1
**IoTNet-3-5**	**0.14**	**89.22%**	**499.7**

**Table 9:** The improvements of the proposed best model for the SVHN data set over the state-of-the-art networks.

Model	Acc Improvement	FLOPs Saving
EffNet V2	1.92%	**58.5%**
MobileNetV2	2.51%	57.03%
EffNet V1	0.71%	3.46%
MobileNet	3.58%	35.39%
ShuffleNet	**6.49%**	31.84%

**Table 10:** Accuracy of the best candidate models found using the multi-filtering search, then trained and tested on GTSRB. The first group contains networks with larger configurations, while the second group comprises smaller ones.

Model	Widening Factor *k*	Mean Acc	kFLOPs
**IoTNet-3-2**	**0.22**	**93.17%**	**531.0**
IoTNet-3-5	0.15	90.57%	531.5
IoTNet-3-3	0.18	91.84%	427.1
IoTNet-3-3	0.15	88.25%	342.1
IoTNet-3-3	0.13	88.72%	323.9
IoTNet-3-1	0.24	73.33%	310.3
**IoTNet-3-2**	**0.18**	**88.82%**	**301.6**

**Table 11:** Evaluation results of the GTSRB data set. The results are grouped by network sizes in FLOPs. The first group contains larger networks, with the second group showing comparatively smaller models.

Model	Widening Factor *k*	Mean Acc	kFLOPs
EffNet V2	0.3	90.4%	704.5
MobileNetV2	0.31	90.74%	710.7
MobileNet	0.23	88.15%	533.0
ShuffleNet	0.23	88.99%	540.7
**IoTNet-3-2**	**0.22**	**93.17%**	**531.0**
EffNet V1	0.15	**91.79%**	344.1
IoTNet-3-2	0.18	88.82%	301.6

**Table 12:** The improvements of the proposed best model for the GTSRB data set over the state-of-the-art networks, grouped by network sizes.

Model	Acc Improvement	FLOPs Saving
EffNet V2	2.77%	24.63%
MobileNetV2	2.43%	**25.28%**
MobileNet	**5.02%**	0.38%
ShuffleNet	4.18%	1.79%
EffNet	−2.97%	12.35%

**Table 13:** Specifications and environmental settings of the desktop PC and Raspberry Pi.

Device	CPU	Memory	Operating System	Library
PC	I7-2600k@4GHz	16 GB	Ubuntu 18.04 LTS	PyTorch 1
Raspberry Pi 3 Model B+	ARM Cortex@1.4GHz	1 GB	Raspbian Buster 4.19	PyTorch 1

**Table 14:** Comparison of time and space required to process one image from a batch of 128 between a PC and Raspberry Pi. Time is reported as the time taken to process one image, in milliseconds.

Model	Widening Factor	Data Set	kFLOPs	Memory (MB)	Pi - Time (ms)	PC - Time (ms)
IoTNet-3-4	0.7	CIFAR-10	9900	392	87.50	0.78
IoTNet-3-2	0.68	CIFAR-10	4200	192	46.09	0.39
IoTNet-3-5	0.14	SVHN	499.7	15	5.94	0.20
IoTNet-3-2	0.22	GTSRB	531.0	27	4.61	0.13
IoTNet-3-2	0.18	GTSRB	301.6	14	4.06	0.16
